# Functional outcomes, extent of resection, and bright/vague fluorescence interface in resection of glioblastomas involving the motor pathways assisted by 5-ALA

**DOI:** 10.1007/s00701-022-05358-9

**Published:** 2022-09-10

**Authors:** Giovanni Muscas, Simone Orlandini, Camilla Bonaudo, Maddalena Dardo, Alice Esposito, Luca Campagnaro, Riccardo Carrai, Enrico Fainardi, Pietro Ciccarino, Alessandro Della Puppa

**Affiliations:** 1 Neurosurgery Clinic, Academic Neurosurgery, Department of Neuroscience, Psychology, Pharmacology and Child Health, Careggi University Hospital and University of Florence, Largo Palagi 1, 50139 Florence, Italy; 2 Neurosurgery Unit, Department of Neuroscience, Padova University Hospital, Padua, Italy; 3 Neurophysiopathology Unit, Careggi University Hospital, Florence, Italy; 4 Neuroradiology Unit, Careggi University Hospital and University of Florence, Florence, Italy

**Keywords:** Glioblastoma, Rolandic, 5-ALA, Extent of resection, Surgical outcome

## Abstract

**Background:**

5-Aminolevulinic acid (5-ALA) fluorescence can maximize perirolandic glioblastoma (GBM) resection with low rates of postoperative sequelae. Our purpose was to present the outcomes of our experience and compare them with other literature reports to investigate the potential influence of different intraoperative monitoring strategies and to evaluate the role of intraoperative data on neurological and radiological outcomes in our series.

**Methods:**

We retrospectively analyzed our prospectively collected database of GBM involving the motor pathways. Each patient underwent tumor exeresis with intraoperative 5-ALA fluorescence visualization. Our monitoring strategy was based on direct stimulation (DS), combined with cortical or transcranial MEPs. The radiological outcome was evaluated with CRET vs. residual tumor, and the neurological outcome as improved, unchanged, or worsened. We also performed a literature review to compare our results with state-of-the-art on the subject.

**Results:**

Sixty-five patients were included. CRET was 63.1%, permanent postoperative impairment was 1.5%, and DS’s lowest motor threshold was 5 mA. In the literature, CRET was 25–73%, permanent postoperative impairment 3–16%, and DS lowest motor threshold was 1–3 mA. Our monitoring strategy identified a motor pathway in 60% of cases in faint fluorescent tissue, and its location in bright/faint fluorescence was predictive of CRET (*p* < 0.001). A preoperative motor deficit was associated with a worse clinical outcome (*p* < 0.001). Resection of bright fluorescent tissue was stopped in 26%, and fluorescence type of residual tumor was associated with higher CRET grades (*p* < 0.001).

**Conclusions:**

Based on the data presented and the current literature, distinct monitoring strategies can achieve different onco-functional outcomes in 5-ALA-guided resection of a glioblastoma (GBM) motor pathway. Intraoperatively, functional and fluorescence data close to a bright/vague interface could be helpful to predict onco-functional outcomes.

## Introduction

Glioblastoma (GBM) prognosis is related to the amount of residual tumor after surgical exeresis [18]. One of the most relevant aspects of surgery for these lesions is achieving a satisfactory extent of tumor resection while preserving highly “functional” brain tissue integrity to avoid invalidating iatrogenic neurological deficits [9, 10, 13, 14]. The concept of maximal safe resection summarizes this and represents the primary goal of surgery for high-grade gliomas [8, 11, 18, 23, 24]. In recent years, to safely accomplish this, several intraoperative procedures, such as tumor visualization of 5-aminolevulinic acid (5-ALA)-associated fluorescence and intraoperative neurophysiological monitoring (IOM) tasks have been developed and established. The results achieved in the extent of resection and surgical morbidity using these techniques have shown an improved outcome, granting these approaches a central role in neurooncological surgery [2, 5, 8, 12, 17]. GBM in critical anatomical regions, such as the Rolandic lobe, precentral and postcentral white matter, or deep nuclei in close anatomical relationship with the cortical-spinal tract, still represent a challenge. In the past, resection of these lesions was rarely attempted, and only recently the techniques mentioned above allowed satisfactory results in terms of neurological outcome and extent of resection [3, 13, 18]. Due to these lesions’ relative rarity and surgical complexity, the use of 5-ALA and IOM and their impact on surgery for GBM involving the motor pathways at different anatomical locations deserves further investigations to expand the surgical indications and possibly select or exclude candidates for safe surgical resection.

Here, we present our surgical experience with GBM involving the motor pathways operated on with intraoperative 5-ALA fluorescence visualization, focusing on motor function (MF) outcomes, the potential role of different IOM strategies, and intraoperative data on bright/fair 5-ALA fluorescence interface. For this purpose, we retrospectively analyzed our experience and data from a comprehensive literature review.

## Material and methods

### Our series

The study was conducted under the regulation of the Local Ethical Board of our Institution, and all patients signed informed consent for prospective clinical data storage and utilization. We retrospectively retrieved data from our prospectively collected database. Data were retrieved concerning demographics, medical history, clinical and radiological presentation (MRI), anatomical location, intraoperative 5-ALA fluorescence visualization, IOM specifics, and clinical and radiological outcomes. Patients with a suspected diagnosis of new or recurrent GBM operated on from June 2016 to June 2019 were included. Further inclusion criteria were age > 18 years, tumor localization in an anatomical location involving the corticospinal tract (less than < 10 mm as reconstructed on preoperative diffusion tensor imaging (DTI) tractography), histological confirmation of GBM, and willingness to provide written informed consent to participate in the study. Sixty-five patients with a mean age of 56.9 (± 10) years met the inclusion criteria, of which 35 (53.8%) were female.

MR scans were acquired preoperatively and at 30 days postoperatively on a 1.5 Tesla (Koninklijke Philips N.V., Amsterdam, Netherlands), including T1-weighted native, T1-weighted with contrast enhancement, T2-weighted, and fluid attenuation inverse recovery (FLAIR) sequences, and the maximal tumor diameter was measured. Each patient underwent 3–5 days of preoperative preparation with dexamethasone 12 mg/24 h. Clinical and demographical data included patients’ age, gender, medical history concerning the previous exeresis of GBM, and the presence of a preoperative motor deficit matching the anatomical location of the lesion after steroid therapy. DTI fiber tracking was performed on the StealthViz™ Planning Station (Medtronic Inc., Dublin, Ireland), utilizing the precentral gyrus and the cerebral peduncles as regions of interest [4] to assess the lesions’ relationship with the motor pathways and fulfill the inclusion criteria. Lesions’ location was dichotomized into precentral (or frontal) and postcentral (or parietal) and further subdivided according to their relationship to the cortical surface as superficial when the cortex was affected, subcortical when the lesion was entirely located in the subcortical white matter, and deep when the basal ganglia were involved.

Preoperative 5-ALA was orally administered 3 h before anesthesia induction at 20 mg/kg (Gliolan®, Medac GmbH, Wedel, Germany), and exposure to direct light was avoided for the following 24 h after administration. Intraoperative 5-ALA fluorescence visualization was accomplished via a Kinevo® 900 microscope equipped with a Blue 400 filter (Carl Zeiss AG, Oberkochen, Germany).

Intraoperative monitoring was performed with direct cortical and subcortical stimulation (DCS/SCS) as stand-alone or in association with trans-scalp stimulation (TSS) to obtain motor evoked potentials (MEP) or a subdural electrodes strip. Muscle MEPs were recorded by pairs of needle electrodes (EBN, Florence, Italy) inserted in the biceps brachialis, extensor digits communis, adductor digit minimum, tibialis anterior, and abductor hallucis brevis of side muscles contralateral to the site of the supra-cortical brain lesion and recorded on a 100-ms epoch length and a band-pass filter of 1.5–853 Hz. Transcranial electric stimulation was performed by corkscrew electrodes (BIONEN, Florence, Italy) placed subcutaneously at C1, C2, according to the international 10–20 system [6]. We used C1/C2 stimulation montage. It is usually less potent and produces less patient movement than C3/C4. A short train (4 pulses) of monopolar, anodal, rectangular pulses with an inter-stimulus interval (ISI) of 2–4 ms [12]. For direct cortical and subcortical stimulation, we used the monopolar multipulse technique with a constant voltage probe stimulator (NATUS Neurol Inc., Middleton, USA). A short train (4–5 pulses) of square-waved pulses was applied at a 200–250-Hz frequency with impulse duration of 0.5 ms. Stimulation intensity was increased stepwise until action potentials were elicited in the target muscles. The maximum was set at 20 mA, and the cathode was located ipsilaterally outside the operating field. We used anodal stimuli, while cathodal stimuli were used for subcortical stimuli. After the primary motor cortex was individuated by direct cortical electric stimulation, an electrode strip (AD-TECH, Oak Creek, USA) (row of four electrodes embedded in silicon) was placed over the cortex when deemed appropriate. We use a needle electrode placed on Fpz, according to the international 10–20 system [6] as reference. First of all, we identified the best electrode of the strip to obtain the best MEP from the upper or lower limb according to the neurosurgical conditions. Then, this electrode was used to monitor the corticospinal pathway. A short train (4–5 pulses) of rectangular pulses was applied at a 200–250-Hz frequency with impulse duration of 0.5 ms. Stimulation intensity was increased stepwise until action potentials were elicited in the target muscles.

Tumor resection included the fluorescent part of the lesion and was interrupted when a positive response on direct cortical stimulation was present at 5-mA intensity or when evoked potentials showed an amplitude reduction of at least 50% from the baseline. Information on the employment of these techniques as stand-alone or in combination was collected.

Concerning the intraoperative 5-ALA fluorescence visualization, information gathered considered the presence of function on direct stimulation at 5 mA (thereby requiring interruption of tumor exeresis) either within non-fluorescent tissue or in 5-ALA positive areas, further subdivided in bright and faint fluorescence. Similarly, the surgeon’s intraoperative perception of complete resection was registered, and the presence of residual tumor was classified as no residual tumor, faint residual fluorescence, or bright fluorescence.

Finally, the neurological outcome was assessed at 30 days postoperatively and classified as improved, unchanged, or worsened (defined as any detectable change on the Medical Research Council (MRC) motor scale). The 30-day radiological outcome was dichotomized as CRET or the presence of a residual tumor.

Continuous variables are presented as mean with standard deviation, nominal variables as frequencies with percentages. To assess heterogeneity in outcomes between the dependent variable subgroups, the *χ*^2^ test for homogeneity was performed on nominal data, and an independent samples *t*-test was performed on continuous data. Additionally, the relationships between each feature and the dependent variable were investigated with a binomial logistic regression for the radiological outcome and a multinomial logistic regression for the neurological outcome. The Fisher exact test assessed the radiological and clinical outcome relationship. Statistical significance set for *p* < 0.05 after Bonferroni correction. All analyses were performed with SPSS Statistics 23 (IBM Corp. Armonk, NY, USA).

### Literature review

We performed a literature review using the Pubmed research engine, including “rolandic glioblastoma,” “motor area glioblastoma,” and “5-ALA glioblastoma” as keywords. Retrospective and prospective cohort studies and reports older than 20 years were included, and letters and case reports were excluded. We also excluded studies with fewer than fifteen patients and those with various histology (e.g., metastases).

## Results

### Our series

A summary of the patients’ features is reported in Table 1. Of all patients, 53 (81.5%) underwent surgical exeresis of a newly diagnosed GBM, whereas the remaining 12 (18.5%) displayed a recurrent tumor, and a preoperative motor deficit was present in 47 (72.3%) patients. Concerning the anatomical location, the lesion involved the frontal/precentral lobe in 42 (64.6%) patients, the parietal postcentral lobe in 23 (35.4%), and was located superficially in 29 (46%) cases, subcortically in 29 (46%), and embedded in the deep white matter in the remaining 7 (8%) patients. Intraoperative monitoring consisted of DCS/SCS as stand-alone in 20 (30.8%) cases, whereas the adjunct of subdural electrode strips or TSS was employed in 29 (44.6%) and 16 (24.6%) patients, respectively. Considering the 5-ALA positivity, the intraoperative monitoring was able to identify motor function in 5-ALA-negative areas in 10 (15.4%) patients, whereas function was detected within areas of bright fluorescence in 16 (24.6%) cases and faint fluorescence in the remaining 39 (60%) patients. Accordingly, the intraoperative perception of tumor resection was complete resection in 7 (10.8%) patients, and in the remaining cases, tumor remnants consisted of 5-ALA bright or faint fluorescence in 17 (26.2%) and 41 (63.1%) patients, respectively (Table 1).

**Table 1:** Summary of the patients’ features

Age	*Mean* ± *St. dev*	56.9 (± 10.0)
Gender	*Male*	30 (46.2%)
	*Female*	35 (53.8%)
New diagnosis vs recurrence	*New diagnosis*	53 (81.5%)
	*Recurrence*	12 (18.5%)
Lesion location	*Precentral/frontal*	42 (64.6%)
	*Postcentral/parietal*	23 (35.4%)
Depth	*Cortical*	29 (46%)
	*Subcortical*	29 (46%)
	*Deep*	7 (8%)
Preoperative motor deficit	*Yes*	47 (72.3%)
	*No*	18 (27.7%)
Max diamter (cm)	*Mean* ± *St. dev*	3.75 (± 1.03)
IOM	*Stimulation alone*	20 (30.8%)
	*Stimulation & strip*	29 (44.6%)
	*Stimulation & TSS*	16 (24.6%)
Detection of function	*No*	10 (15.4%)
	*Bright*	16 (24.6%)
	*Faint*	39 (60%)
Radicality of resection	*No*	7 (10.8%)
	*Yes*, *bright*	17 (26.2%)
	*Yes*, *faint*	41 (63.1%)

CRET was achieved in 41 (63.1%) patients, while a residual was radiologically detected in 24 (36.9%). In all cases, GTR > 90% was achieved. CRET (Table 2) was higher, close to statistical significance when correcting for multiple comparisons (*p* = 0.006), in newly diagnosed than in recurrent GBM, in postcentral and superficial tumors, and when patients had no preoperative motor impairment. Again, CRET was statistically associated with no residual bright fluorescent tumor detected in the surgical cavity, while a faint fluorescent tumor left was in up to 87% of cases not reported on post-op MRI. Detection of function into o close to the bright fluorescent tumor was statistically associated with a lower CRET (*p* < 0.001).

**Table 2:** Proportions of investigated features and different radiological outcomes

		Post-op MR		
		CRET	Residual	
		41 (63.1%)	24 (36.9%)	
Age	*Mean* ± *St. dev*	57.6 (± 10.3)	55.9 (± 8.8)	*p* = 0.5
Gender	*Male*	20 (69.0%)	10 (31.0%)	*p* = 0.48
	*Female*	20 (57.1%)	15 (42.9%)	
New diagnosis vs recurrence	*New diagnosis*	38 (71.7%)	15 (28.3%)	*p* = 0.006
	*Recurrence*	3 (25.0%)	9 (75.0%)	
Lesion location	*Precentral/frontal*	24 (57.1%)	18 (42.9%)	*p* = 0.14
	*Postcentral/parietal*	17 (73.9%)	6 (26.1%)	
Depth	*Cortical*	19 (65.5%)	10 (34.5%)	*p* = 0.57
	*Subortical*	19 (65.5%)	10 (34.5%)	
	*Deep*	3 (40.0%)	4 (60.0%)	
Preoperative motor deficit	*Yes*	27 (57.4%)	20 (42.6%)	*p* = 0.16
	*No*	14 (77.8%)	4 (22.2%)	
Max diameter (cm)	*Mean* ± *St. dev*	3.59 (± 0.95)	4.04 (± 1.1)	*p* = 0.85
IOM	*Stimulation alone*	15 (75%)	5 (25%)	*p* = 0.41
	*Stimulation & strip*	17 (58.6%)	12 (41.4%)	
	*Stimulation & TSS*	9 (56.3%)	7 (43.7%)	
Detection of function	*No*	8 (80.0%)	2 (20.0%)	***p* < 0.001**
	*Bright*	0 (0.0%)	16 (100%)	
	*Faint*	33 (80.5%)	6 (29.5%)	
Radicality of resection	*No*	5 (71.4%)	2 (28.6%)	***p* < 0.001**
	*Yes*, *bright*	0 (0.0%)	17 (100%)	
	*Yes*, *faint*	36 (87.8%)	5 (12.2%)	

The preoperative neurological status was unchanged postoperatively in 28 (43.1%) patients, improved in 36 (55.4%), and worsened in 1 (1.5%) patients (Tables 2 and 3). The motor outcome did not depend on tumor location, depth, or recurrent surgery. Conversely, it was statistically associated with preoperative motor status (*p* < 0.001). In the only case with a postoperative impairment reported intraoperatively, motor function was detected in faint fluorescence tissue.

**Table 3:** Proportions of investigated features and different radiological outcomes

		Neurological outcome			
		Unchanged	Improved	Worsened	
		28 (43.1%)	36 (55.4%)	1 (1.5%)	
Age	*Mean* ± *St. dev*	58.8 (± 8.6)	54.5 (± 10.9)	52	*p* = 0.53
Gender	*Male*	8 (27.6%)	21 (72.4%)	0 (0.05)	*p* = 0.03
	*Female*	19 54.3%)	15 (42.9%)	1 (2.9%)	
New diagnosis vs recurrence	*New diagnosis*	26 (49.1%)	26 (49.1%)	1 (1.8%)	*p* = 0.78
	*Recurrence*	2 (16.7%)	10 (83.3%)	0 (0.0%)	
Lesion location	*Precentral/frontal*	19 (45.2%)	22 (52.4%)	1 (2.4%)	*p* = 0.75
	*Postcentral/parietal*	9 (39.1%)	14 (60.9%)	0 (0.0%)	
Depth	*Cortical*	12 (41.4%)	16 (55.2%)	1 (3.4%)	*p* = 1
	*Subortical*	13 (44.8%)	16% (55.2%)	0 (0.0%)	
	*Deep*	3 (40.0%)	4 (60.0%)	0 (0.0%)	
Preoperative motor deficit	*Yes*	28 (59.6%)	18 (38.3%)	1 (2.1%)	*p* < 0.001
	*No*	18 (100.0%)	0 (0%)	0 (0.0%)	
Max diameter (cm)	*Mean* ± *St. dev*	3.89 (± 0.87)	3.64 (± 1.1)	4	*p* = 0.45
IOM	*Stimulation alone*	12 (60.0%)	7 (35.0%)	1 (5.0%)	*p* = 0.12
	*Stimulation & strip*	11 (37.9%)	18 (62.1%	0 (0.0%)	
	*Stimulation & TSS*	5 (31.3%)	11 (68.7%)	0 (0.0%)	
Detection of function	*No*	4 (40%)	6 (60.0%)	0 (0.0%)	*p* = 0.73
	*Bright*	3 (18.8%)	13 (81.3%)	0 (0.0%)	
	*Faint*	21 (53.8%)	17 (43.6%)	1 (2.6%)	
Radicality of resection	*No*	3 (42.9%)	4 (57.1%)	0 (0.0%)	*p* = 0.59
	*Yes*, *bright*	3 (17.6%)	14 (82.4%)	0 (0.0%)	
	*Yes*, *faint*	22 (53.7%)	18 (43.9%)	1 (2.4%)	

### Literature review

A total of 12 studies matched the inclusion criteria (see Table 4). Only one focused explicitly on GBM surgery guided by 5-ALA in the motor pathway [18]. The remaining series included glioma patients with grades other than IV or tumor locations other than motor pathways. Schucht et al. reported their experience on 67 consecutive patients operated on with a specific strategy based on continuous dynamic monopolar motor mapping (short-train inter-stimulus interval 4.0 ms, pulse duration 500 ms) coupled to an acoustic MEP alarm. CRET was 73%, and postoperative motor impairment was 30% immediately after surgery and 4% at three months evaluation of patients. The lowest motor threshold ranged from > 20 to 1 mA. Even though this study is the only one focusing on GBM surgery involving the motor pathways with the assistance of 5-ALA, it must be emphasized that it involved a specific technique not routinely applied in the neurosurgical community.

**Table 4:** Previous studies dealing with high-grade gliomas (HGG) involving the motor pathways (*DCS*, direct cortical (and subcortical) stimulation; *CRET*, complete resection of the contrast-enhancing part of the tumor; *GTR*, gross total resection)

Author	N. of patients	Intraoperative monitoring technique	Awake surgery	Radicality	Postoperative neurological impairment	3-month permanent neurological impairment
Stummer et al. (2000)^16^	52	NA	No	63% CRET	6%	2%
Feigl et al. (2010)^17^	18	Evoked potentials	No	64% GTR	11%	NA
Diez Valle et al. (2011)^18^	36	NA	No	83.3% GTR	25%	NA
Bogosavljevic et al. (2012)^19^	26	Cortical strip electrodes	No	17% GTR	NA	NA
Della Puppa et al. (2013)^20^	31	Evoked potentials; DCS	Yes	74% CRET	10%	2.5%
Schucht et al. (2012)^13^	103	Evoked potentials: DCS	Yes	89% CRET	NA	7.5%
Aldave et al. (2013)^21^	118	NA	No	73% CRET	NA	NA
Schucht et al. (2014)^1^	72	Continuous monopolar motor mapping	No	73% CRET	30%	4%
Kim et al. (2014)^22^	80	Na	No	80% GTR	25%	7.5%
Noell et al. (2015)^23^	29	Evoked potentials; DCS	No	25% CRET	17%	16%
Picht et al. (2016)^24^	127	Evoked potentials; DCS	No	45–61% GTR	26%	15%
Picart et al. (2017)^25^	51	NA	No	NA	16%	4%

A further study by Noell and colleagues focused on gliomas involving the motor patwhays [15] reported 29 patients with a CRET of 25% and permanent postoperative impairment of 16%. The monitoring strategy was based on a bipolar probe with the tips 5 mm apart. Pulses for cortical and subcortical stimulations were rectangular, and the current was biphasic with a frequency of 60 Hz. The current intensity ranged from 1 to 6 mA. However, grade III and IV gliomas were enrolled, pooling the results. Moreover, as mentioned above, 5-ALA was not employed.

The remaining studies displayed heterogeneity concerning intraoperative neurophysiological monitoring strategy, tumor histology (i.e., grading), and anatomical location, and only a proportion of patients were affected by gliomas in eloquent or, more specifically, motor areas. Overall, CRET ranged from 72 to 89%, but when focusing on eloquent areas was 64–74%, and postoperative impairment was 3–17%. Different monitoring strategies with different DCS stimulation lowest motor thresholds were reported (6–3 mA).

## Discussion

The first goal of our study was to characterize the surgical outcome achievable in GBM involving the motor pathways and highlight the role of different intraoperative monitoring techniques and visualization of 5-ALA-associated fluorescence. Our study presents original and interesting data about the role of intraoperative information available when operating with 5-ALA assistance on motor pathway GBM. If confirmed by a more extensive series, these data could be helpful for the intraoperative prediction of both CRET and motor outcome, thus calibrating surgical strategy.

High-grade gliomas in this anatomical location have recently deserved attention due to the high risk of postoperative neurological impairment and unsatisfactory surgical results reported in the past [7]. According to our experience, combining intraoperative 5-ALA fluorescence visualization and neurophysiologic monitoring allows for excellent results regarding postoperative neurological status and recurrence rates at one month (see Tables 2 and 3). Reports on the results of high-grade glioma surgery have recently become more frequent, in parallel with the affirmation of intraoperative monitoring and mapping techniques as an effective adjunct to performing satisfactory resection with tolerable postoperative morbidity rates [1, 16, 25]. Even though intraoperative monitoring and mapping can guide the surgeon to perform an effective and safe tumor removal, a subtotal resection could result from functional tissue embedded within the tumor and incomplete visualization of tumor infiltration. While the former point is an intrinsic aspect of intraoperative monitoring and prevents the onset of a disabling complication, visualization with 5-ALA-associated fluorescence can overcome the latter. The benefit of 5-ALA in terms of the extent of resection and progression-free survival has been proven [21]. Still, indiscriminate removal of 5-ALA fluorescent tissue may lead to higher postoperative deficits, as fluorescence can be observed up to 10 mm beyond the contrast-enhancing part of the lesion on preoperative MR, thereby endangering relevant structures [19]. This is confirmed by the higher rates of postoperative deficits in patients operated with 5-ALA fluorescence compared to white light microscopy [21]. Additionally, the meaning of different fluorescence intensity and the presence of functional tissue within different degrees of fluorescence still deserves further investigation. Therefore, approaching these lesions with the combination of these two adjuncts has been attempted with satisfactory results [18].

Our data show that a standard monitoring strategy based on direct cortical and subcortical stimulation combined with motor evoked potentials can achieve an excellent onco-functional balance with a high CRET and optimal motor outcome. No reports in the literature focused on GBM surgery of the motor pathway assisted by 5-ALA with our monitoring strategy, which is probably the most frequently used in the operating rooms. The only experience reported was Schucht et al. [18], who used an innovative technique based on a continuous dynamic monopolar motor mapping. A worse motor outcome but a better CRET was reported in that series. However, different percentages of recurrent tumors, preoperative deficit (72% in our series vs. 32% of Schuct et al. [18]), data about the distance between tumor and motor pathway (difference in the two series about the percentage of patients undergoing preoperative DTI) represent the main limitation for a formal comparison. Conversely, the higher postoperative motor outcome in our series (1.5% permanent impairment, 55.4% improvement) may depend on the different DS lowest motor threshold in our series (5 mA) in comparison to other (1–3 mmA).

Concerning the extent of resection, diverse CRET rates were associated with different patterns of fluorescence of tumor left; in particular, the residual bright fluorescent tumor was predictive of no radicality of resection, while the absence of bright fluorescent tissue left was indicative of complete removal of contrast-enhancing tumor (*p* < 0.001). Our series confirms data reported by Stummer et al. in a minor series in the past [20], and 5-ALA-assisted surgery of GBM enables the surgeon to achieve CRET by removing bright fluorescence and pursuing supramarginal resection by removing faint fluorescence.

The bright/vague interface data about motor/functional data in the bright/vague interface are original. We found motor function more frequently in vague fluorescent tissue (60%) than into or immediately close to bright fluorescent tissue (24%). This probably means bright tumoral components usually displace the motor pathway into soft peritumoral infiltrated tissue. These data can partially explain the postoperative worsening of the patient in which resection was driven into faint tissue.

Attention must be paid to removing faint fluorescent tissue for the higher probability of injury function when operating close to the motor pathway. The DS lowest motor threshold value used during resection could be crucial for faint fluorescence.

Similar to our study, different experiences reported in the literature reported interrupting the tumor exeresis despite persisting 5-ALA fluorescence due to a positive neurophysiological response in 16 patients (24%), of which 9 (56%) still had a CRET. However, no distinction was provided on the entity of fluorescence (i.e., faint versus bright), an aspect that has a relevant role in predicting the presence of tumoral infiltrating cells [22].

Limitations inherent to this study concern its retrospective nature, despite prospectively acquiring data. Additionally, measures of surgical radicality such as volumetric analysis or gross total resection could offer a more precise depiction of the surgical radicality. However, these are subjected to more significant interrater variations, while CRET is a more straightforward, objective, and reproducible criterion, despite being less sensitive.

Despite injuries in the SMA being clinically associated with motor deficits indistinguishable from motor pathways’ lesions, the deficit’s pathophysiology and the intra- and postoperative neurophysiological findings differ from pure Rolandic injuries. The function of the SMA is yet to be fully clarified. Moreover, the prognosis could be dramatically different, with SMA lesions typically improving over days to weeks, while motor pathways lesions are potentially permanent. We opted to exclude patients in which this location was involved for these reasons, and we are confident that the SMA’s involvement may have played a limited role in the presence of neurological deficit. Moreover, we did not perform a stratification between left vs. right hemispheric lesion localization: no specific language deficits were encountered concerning postoperative language deficits, and transitory dysarthrias were considered correlated with motor issues. Therefore, such stratification was deemed unnecessary.

Finally, we did not find any statistical relevance concerning CRET and postoperative deficit between newly diagnosed and recurrent tumors. Still, challenges rising in the surgical treatment of the latter are known among neurosurgeons, and their more infiltrative and diffuse nature could present specific challenges that need to be further elucidated when dealing with Rolandic glioblastomas.

## Conclusions

Our study confirms that 5-ALA-assisted surgery of GBM enables the surgeon to achieve CRET by removing bright fluorescence and pursuing supramarginal resection by removing faint fluorescence. Based on the literature review and our data, in 5-ALA-guided resection of a motor pathway GBM, distinct monitoring strategies can achieve different onco-functional outcomes. Intraoperatively, functional, and fluorescence data close to a bright/vague interface can help predict onco-functional outcomes. When operating close to the motor pathway, attention must be paid to removing faint fluorescent tissue for the higher probability of injury function. The DS lowest motor threshold value used during resection could be crucial for faint fluorescence.

## Abbreviations

5-ALA: 5-Aminolevulinic acid
CRET: Complete resection of the enhancing part of the tumor
DCS/SCS: Direct cortical and subcortical stimulation
DTI: Diffusion tensor imaging
FLAIR: Fluid attenuation inverse recovery
GTR: Gross total resection
HGG: High-grade gliomas
IOM: Intraoperative neurophysiological monitoring/intraoperative monitoring
GBM: Glioblastoma
mA: Milliampere
MEP: Motor evoked potential
MF: Motor function
MRC: Medical Research Council
MRI: Magnetic resonance imaging
TSS: Trans-scalp stimulation
